# Differential ability of MSCs isolated from placenta and cord as feeders for supporting *ex vivo* expansion of umbilical cord blood derived CD34^+^ cells

**DOI:** 10.1186/s13287-015-0194-y

**Published:** 2015-10-19

**Authors:** Darshana Kadekar, Vaijayanti Kale, Lalita Limaye

**Affiliations:** Stem Cell Laboratory, National Centre for Cell Science, University of Pune Campus, Ganeshkhind, Pune, 411007 Maharashtra India

**Keywords:** *Ex vivo* HSC expansion, Cord mesenchymal stem cells, Placental mesenchymal stem cells

## Abstract

**Introduction:**

*Ex vivo* expansion of umbilical cord blood (UCB) is attempted to increase cell numbers to overcome the limitation of cell dose. Presently, suspension cultures or feeder mediated co-cultures are performed for expansion of hematopoietic stem cells (HSCs). Mesenchymal stem cells (MSCs) have proved to be efficient feeders for the maintenance of HSCs. Here, we have established MSCs-HSCs co-culture system with MSCs isolated from less invasive and ethically acceptable sources like umbilical cord tissue (C-MSCs) and placenta (P-MSCs). MSCs derived from these tissues are often compared with bone marrow derived MSCs (BM-MSCs) which are considered as a gold standard. However, so far none of the studies have directly compared C-MSCs with P-MSCs as feeders for *ex vivo* expansion of HSCs. Thus, we for the first time performed a systematic comparison of hematopoietic supportive capability of C and P-MSCs using paired samples.

**Methods:**

UCB-derived CD34^+^ cells were isolated and co-cultured on irradiated C and P-MSCs for 10 days. C-MSCs and P-MSCs were isolated from the same donor. The cultures comprised of serum-free medium supplemented with 25 ng/ml each of SCF, TPO, Flt-3 L and IL-6. After 10 days cells were collected and analyzed for phenotype and functionality.

**Results:**

C-MSCs and P-MSCs were found to be morphologically and phenotypically similar but exhibited differential ability to support *ex vivo* hematopoiesis. Cells expanded on P-MSCs showed higher percentage of primitive cells (CD34^+^CD38^−^), CFU (Colony forming unit) content and LTC-IC (Long term culture initiating cells) ability. CD34^+^ cells expanded on P-MSCs also exhibited better *in vitro* adhesion to fibronectin and migration towards SDF-1α and enhanced NOD/SCID repopulation ability, as compared to those grown on C-MSCs. P-MSCs were found to be closer to BM-MSCs in their ability to expand HSCs. P-MSCs supported expansion of functionally superior HSCs by virtue of reduction in apoptosis of primitive HSCs, higher Wnt and Notch activity, HGF secretion and cell-cell contact. On the other hand, C-MSCs facilitated expansion of progenitors (CD34^+^CD38^+^) and differentiated (CD34^−^CD38^+^) cells by secretion of IL1-α, β, MCP-2, 3 and MIP-3α.

**Conclusions:**

P-MSCs were found to be better feeders for *ex vivo* maintenance of primitive HSCs with higher engraftment potential than the cells expanded with C-MSCs as feeders.

**Electronic supplementary material:**

The online version of this article (doi:10.1186/s13287-015-0194-y) contains supplementary material, which is available to authorized users.

## Introduction

Over the past two decades, umbilical cord blood (UCB) has developed into an attractive and alternative source of hematopoietic stem cells (HSCs) both in clinics and in research. However, insufficient numbers of HSCs in the UCB limits its application in adults as an allogeneic source of HSCs for the transplantation [1]. The limited cell dose can be enhanced either by performing double CB transplantation (DCBT) or by *ex vivo* expansion of UCB. *Ex vivo* expansion stands out to be the most convenient option over the DCBT since, in the latter, there is successful engraftment of only a single CB unit with an elevated risk of graft versus host disease [1–4].

Currently, HSCs are expanded in the presence of a combination of cytokines [4–6]. However, under *in vivo* conditions, HSCs are reliant upon the cytokines and also on the varied components from their niche, such as mesenchymal stem cells (MSCs), endothelial cells, osteoblasts, etc., and extra cellular matrix for their maintenance and differentiation [7]. This emphasizes the need for an optimized culture system which closely resembles the *in vivo* niche and supports the growth of HSCs *in vitro*. Various cell types from the niche are used for the expansion of HSCs, but MSCs in particular are found to be efficient in sustaining the *ex vivo* expansion of HSCs [8–11].

Although BM remains the main source of MSCs, here we have established MSCs-HSCs co-cultures with MSCs isolated from non-invasive sources, such as umbilical cord and placenta [12]. It is reported that MSCs obtained from these sources are morphologically and phenotypically comparable with BM-MSCs [13, 14]. C-MSCs may be situated as an alternative to BM-MSCs in the field of HSCs transplantation as opposed to P-MSCs which are primarily explored as a valuable source for cell replacement therapies. Despite intensive investigation, to the best of our knowledge no report has directly compared the HSCs supportive function of these two stromal populations. We report here a unique observation that C-MSCs and P-MSCs have differential propensities for the maintenance of HSCs. To decipher the basis of the differential ability of these feeders to support the *ex vivo* maintenance and propagation of HSCs we isolated C-MSCs and P-MSCs from the same donor.

We demonstrate here that P-MSCs make better feeders than C-MSCs, and were found to possess similar potential as BM-MSCs for expansion of primitive UCB HSCs. Conversely, expansion mediated by C-MSCs was primarily dependent on the pro-inflammatory cytokines secreted by them yielding differentiated cells. We also report that the differences observed are a reflection of not only the prominent activation of Wnt and Notch signals but also of improved survival signals from P-MSCs as opposed to C-MSCs. Based on all the findings, we conclude that P-MSCs are the most suitable feeders for the *ex vivo* maintenance of functional HSCs.

## Methods

### Ethical approvals for human samples and animal experiments

UCB samples, placenta, and cord were collected from local hospitals after obtaining informed consent with the compliance of the institutional review board (IEC-Institutional ethical committee –NCCS and IC-SCR –Institutional Committee for Stem Cell Research, NCCS) according to the Declaration of Helsinki. Consenting procedures were also approved by the NCCS-IC-SCR- Institutional Committee for Stem Cell Research, NCCS.

Protocols for animal experiments were approved and in accordance with the IAEC-NCCS Institutional Animal Ethical Committee-NCCS/CPCSEA- Committee for the Purpose of Control and Supervision of Experiments on Animals. (Approval number: IACUC-Institutional Animal Care and Use Committee, EAF-Experimental Animal Facility/2004/B-71(III)). Throughout the experiments animals were under the supervision of trained personnel.

### Collection of umbilical cord blood and isolation of CD34+ cells

Cord blood samples were collected from local hospitals after obtaining informed consent with the compliance of the institutional review board (IEC-Institutional ethical committee and IC-SCRT –Institutional Committee for Stem Cell Research, NCCS) according to the Declaration of Helsinki. Consenting procedures were also approved by the IC-SCR- Institutional Committee for Stem Cell Research, NCCS. Mononuclear cells were isolated by the ficoll hypaque density gradient method (density 1.077 g/ml, Sigma Aldrich, St. Louis MO, USA). CD34+ cells were isolated by the positive selection method using Dynal beads according to the manufacturer’s instructions (Dynabeads M-450 CD34; Dynal, ASA, Oslo, Norway).

### Isolation of mesenchymal stem cells from cord tissue and placenta

Umbilical cord and placenta were collected from full term deliveries after obtaining informed consent in accordance with the IEC-Institutional ethical committee and IC-SCRT- Institutional Committee for Stem Cell Research, NCCS. The umbilical cord and a central piece from the placenta were used to isolate the mesenchymal stem cells. The detailed procedure is explained in Ref [15]. Cells between passages three to six were used for the experiments after carrying out the phenotypic characterization of the MSCs. Phenotypic characterization and differentiation to osteoblasts and adipocytes and chondrocytes was carried out as explained in [15].

### Isolation of mesenchymal stem cells from bone marrow

BM-MSCs were a kind gift from Dr.Mohan Wani, NCCS (Pune). BM-MSCs were cultured in RPMI + 20 % Mesen FBS (GIBCO, Grand Island, NY, USA). Phenotypic characterization and differentiation to osteoblasts and adipocytes was carried out as explained earlier [15]. Chondrogenic differentiation was carried out using a StemPro Chondrogenesis kit (Invitrogen, Grand Island, NY, USA) according to the manufacturer’s instructions.

### Establishment of co-cultures of MSCs and HSCs in serum-free medium

MSCs were seeded in 24-well plates (BD Falcon, San Jose, CA, USA) at a cell density of 10^4^ cells/well and were grown until confluence. The monolayers were then irradiated at 8,000 rads and 5 × 10^4^-1 × 10^5^ UCB CD 34^+^ cells in Stem Pro Serum Free medium (GIBCO) were seeded onto the monolayers for contact cultures. For non-contact cultures, 10^4^ cells were seeded on 3 μm cell culture inserts (Millicell, Millipore, San Diego, USA). The co-cultures were incubated for ten days in the presence of a growth factor cocktail containing IL-6, SCF, TPO, and Flt-3-L. All growth factors (Peprotech Inc., Rocky Hill, NJ, USA) were used at a final concentration of 25 ng/ml. After ten days both the non-adherent and adherent fractions were collected and studied for various parameters such as phenotypic characterization, *in vitro* functionality assessment, and *in vivo* engraftment studies in NOD/SCID mice. Assessment of these parameters was carried out using independent cord blood samples.

### Flow cytometry analysis

The expanded cells were subjected to phenotypic characterization using a panel of anti-bodies. For the detection of intracellular proteins the cells were permeabilized using a BD fixperm kit (BD Pharmingen, San Jose, CA, USA) and the staining was done according to the manufacturer’s instructions. Isotype matched antibodies were used as controls. The fluorescently labeled cells were acquired on FACS Canto II and Aria (BD, San Jose, CA, USA) and data were analyzed by FACS DIVA - version 5.0 The details of the antibodies used are as follows: annexin V FITC, CD34 APC/PE, CD38 FITC, Bcl-2 PE, Bax FITC, CD33 FITC/PE, CD19 APC CD3 FITC, CD61 APC, CD45 PE/PE-CY-7 murine CD45.1 Pacific blue (BD Pharmingen) and CD133 PE (Milteny Biotech, Colonge, Germany).

### Colony-forming unit (CFU) assay

Expanded cells (2 × 10^4^) were cultured in 1 % methylcellulose (Sigma Aldrich) with a combination of the growth factors: SCF 20 ng/mL, GM-CSF 2 ng/mL, IL-3 4 ng/mL, and Epo 2 U/mL. The plates were incubated under humidified conditions at 37 °C, in 5 % CO2 for 14 days. Based on the differential morphology of the colonies they were scored as blast-forming unit erythroid (BFU-E), granulocyte-monocyte (GM), granulocyte-erythroid-monocyte-megakaryocyte (GEMM), and megakaryocytes (MK).

### Long-term culture initiating cell (LTC-IC) assay

M210B4 (murine stromal cell line, ATCC) cells was grown on collagen-coated wells of 24-well plates to confluency. The plates were gamma-irradiated (8,000 rads) and 1 × 10^5^ expanded cells were seeded on feeder layers in human myelocult medium (Stem Cell Technology, Vancouver, Canada) supplemented with hydrocortisone (10^−6^ M). The cultures were maintained for four to eight weeks with weekly demi-defoliation. LTC-IC activity was assessed by the CFU assay.

### *In vitro* migration towards SDF1α

Expanded cells (1 × 10^5^) were seeded in the upper chamber of a transwell migration set up (8 μ BD Falcon). A total of 100 ng of SDF 1α was added to the lower wells containing 600 ml of medium (IMDM + 2 % FCS). One set was run without the addition of SDF1α as a control to assess spontaneous migration. The cells were allowed to migrate for five h at 37 °C. The migrated cells were collected, manually counted and a graph of total migrated cells in both sets was plotted after subtracting the number of spontaneously migrated cells from the total number of migrated cells. Further, the migrated fraction was analyzed to check the CD34^+^CD38^−^ content.

### Cell – matrix adhesion assay

Adhesive capacities of the expanded cells were evaluated by determining their adhesion to fibronectin. The CD34+ cells were sort purified from the expanded cells and 10^5^ cells were seeded on fibronectin-coated 96-well plates. The plates were incubated for 35 min (37 °C, 5 % CO2). Unbound cells were gently washed off and the adhered cells were fixed using 4 % paraformaldehyde (ten min, ambient temperature) and stained using crystal violet (0.1 % in 70 % methanol). The plates were washed three times using sterile distilled water to remove the excess stain and the cells were then lysed with Triton X 100 (30 min, ambient temperature) to release the crystal violet dye. The absorbance was measured at 550 nm. A higher absorbance corresponded with more adhesion.

### NOD/SCID repopulation assay

The NOD/LtSZ-scid/scid mice (Jackson Laboratory, Bar Harbor, ME, USA) were bred in the animal facility of NCCS. All the protocols were approved and in accordance with IAEC-NCCS Institutional animal ethical committee -NCCS/CPCSEA- Committee for the Purpose of Control and Supervision of Experiments on Animals.(Approval number: IACUC-Institutional Animal Care and Use Committee, EAF-Experimental Animal Facility/2004/B-71(III)). Throughout the experiments animals were under the supervision of trained personnel. Mice at four to six weeks of age were exposed to a sub-lethal dose of 300 rads total body irradiation from a ^60^Co source (Gammachamber5000, BRIT, Navi Mumbai, India). Expanded cells (10^6^) from C- and P-MSCs sets were then infused through the tail vein into the sub-lethally irradiated mice (*n* = 5 – 10). Short-term and long-term engraftment was assessed in bone marrow and spleen at the 4th and 12th weeks after infusion by probing human CD45^+^ cells against the host murine CD45 background. The presence of greater than 0.1 % in the total nucleated cells of the recipient was considered as a successful engraftment. The multi-lineage engraftment from the donor was determined using a panel of antibodies against the following markers: CD34^+^ (stem cell marker), CD33 (myeloid cells), CD3, CD19, and CD56 (lymphoid cells). The antibodies were purchased from BD Pharmingen. The respective isotype antibodies (Beckton Dickinson, San Jose, CA, USA) were included as control. For staining of peripheral blood, 100 μl of blood was incubated with the antibodies, further RBCs were lysed with lysis buffer (BD) and washed twice with PBS containing 0.1 % BSA. A minimum of 1,00,000 events were acquired on FACS Canto II (BD).

### Apoptosis detection by annexin V/PI assay

After ten days of expansion, cells were harvested and 10^5^ cells were taken for annexinV/PI staining. Briefly, the cells were first washed with PBS and re-suspended in 1 × binding buffer. A total of 5 μL of annexin V FITC (BD Biosciences) was added and incubated at room temperature for 20 min. Cells were again washed with 1 × binding buffer to remove the excess of antibody. Prior to acquisition on flow cytometer, 0.5 μg of propidium iodide was added to each tube and then the cells were analyzed by flow cytometry.

### Wnt and Notch signaling in MSCs-HSCs co-cultures by western blot

P- and C-MSCs were irradiated and cell lysates were prepared after 96 h of irradiation in RIPA buffer. For expanded cells, CD34+ cells were sorted after expansion and the cell lysate was prepared in RIPA buffer. A total of 30 μg of protein was loaded on 10 % SDS-PAGE. Protein samples were then transferred to a PVDF membrane and overnight blocking with 5 % BSA in TBST was carried out. For detection, the panel of antibodies listed below (Cell Signaling Technology) was used. For detection, a chemiluminescent based detection kit from Cell Signaling Technology was used. Densitometric analysis was performed using Image J software and each protein was normalized to the respective β-actin levels. Primary antibodies were: β-catenin (D10AB) XP rabbit mAB, phospho β-catenin (Ser33/37/Thr41), phospho β- catenin (Ser552), TCF-1 (C63D9) LEF1 (C12A5), Dll-1, jagged-1, notch1(D6F11), cleaved notch 1 (NICD V1744) (D3BB), Bmi-1 and Hes. Secondary antibodies were: anti-rabbit IgG HRP-linked Ab and anti-Mouse IgG HRP-linked Ab.

### Cytokine array

Cytokines and chemokines present in the CMs were measured using the human Cytokine Array C5 (AAH-CYT-5-8) following the manufacturer’s instructions (Ray Biotech, Norcross GA, USA). Densitometry analyses were performed using Image J software. All values were normalized according to the mean intensity of positive and negative controls. Fold change was calculated according to the manufacturer’s instructions.

### Statistical analysis

The statistical differences between groups were analyzed by one way repeated measure analysis of variance using the software SIGMA STAT (Jandel Scientific Corporation, San Rafael, CA, USA) for all the experiments. The values were plotted as mean ± standard deviation Probability values *p ≤ 0.05, **p ≤ 0.01, and ***p ≤ 0.001 were considered statistically significant.

## Results

### C- and P-MSCs share similar morphology, phenotype and differentiation potential towards osteoblasts, adipocytes and chondrocytes

**Fig. 1:**
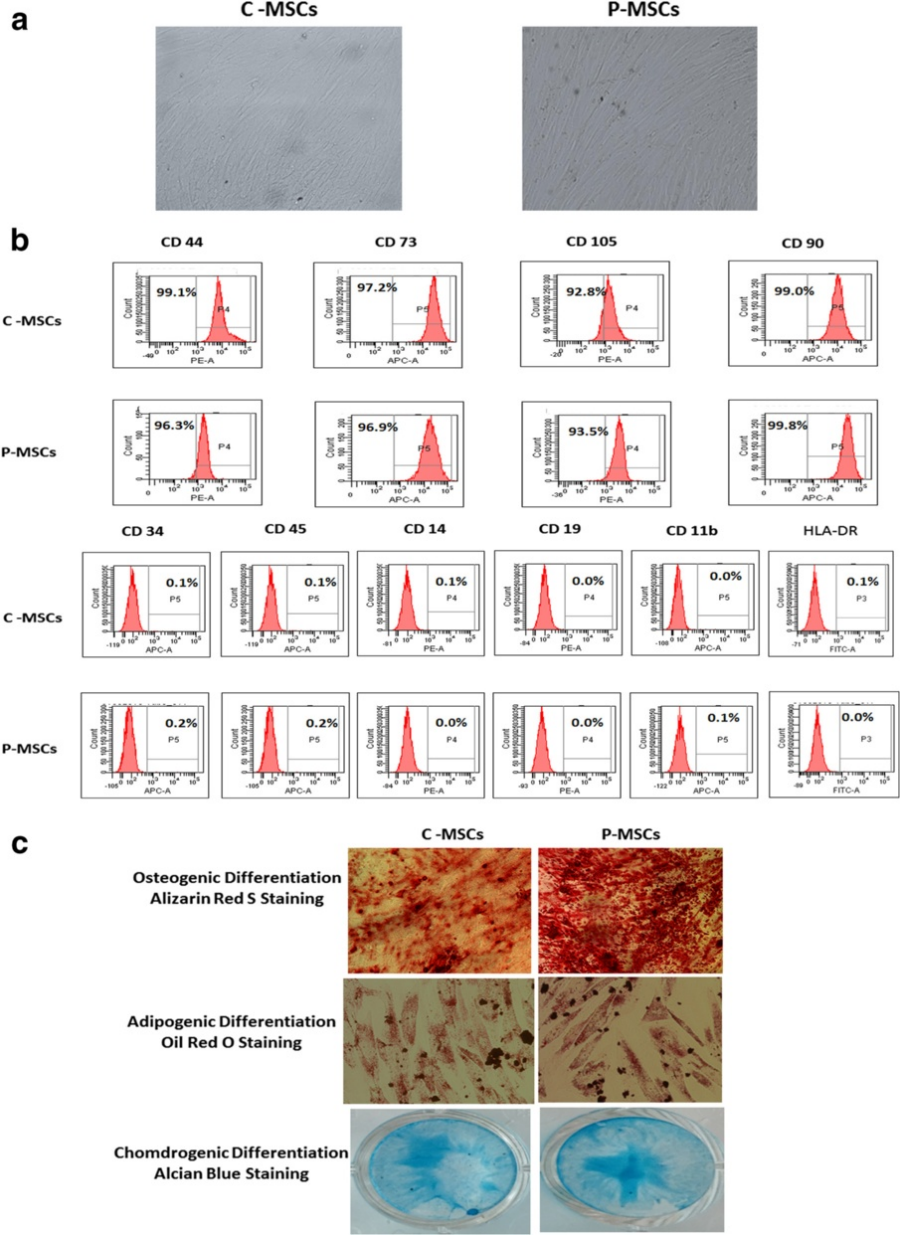
C- and P-MSCs displayed similar morphology and immuno-phenotype. **a** Fibroblastic morphology exhibited by C- and P-MSCs as seen under phase contrast microscope (10X). **b** Histogram profile of representative samples of C- and P-MSCs exhibiting expression of markers such as CD44, CD90, CD73, and CD105. No expression was found for CD45, CD34, CD14, CD19, CD11b, and HLA-DR. **c** *Upper panel* represents osteogenic differentiation of MSCs by staining with alizarin red S. *Middle panel* is for adipogenic differentiation confirmed after lipid droplets stained by oil red o. Chondrogenic differentiation of C- and P-MSCs confirmed with Alcian blue stain. C*-MSCs* cord-derived mesenchymal stem cells, P*-MSCs* placenta-derived mesenchymal stem cells

First, we isolated MSCs from cord (C-MSCs) and placenta (P-MSCs). C- and P-MSCs had similar fibroblastic morphologya (Fig. 1a). C- and P-MSCs showed similar marker expression of molecules such as CD44, CD73, CD105 and CD90, and were found to be negative for CD34, CD45, CD14, CD19, CD11b and HLA-DR which is in accordance with the International Society for Cellular Therapy criteria for defining MSCs [16] (Fig. 1b). Furthermore, we evaluated their multi-lineage differentiation potential by inducing them to differentiate into osteoblasts, adipocytes, and chondrocytes. We found that C- and P-MSCs could be differentiated into all three lineages (Fig. 1c).

### P-MSCs were superior to C-MSCs and closer to BM-MSCs for the expansion of primitive HSCs

We next compared C-MSCs and P-MSCs for their ability to support hematopoiesis. Initially we used C-MSCs and P-MSCs isolated from different donors. As we observed striking differences in the expansion efficiency of the two MSCs (data not shown), expansion was attempted using C- and P-MSCs from paired samples.

**Fig. 2:**
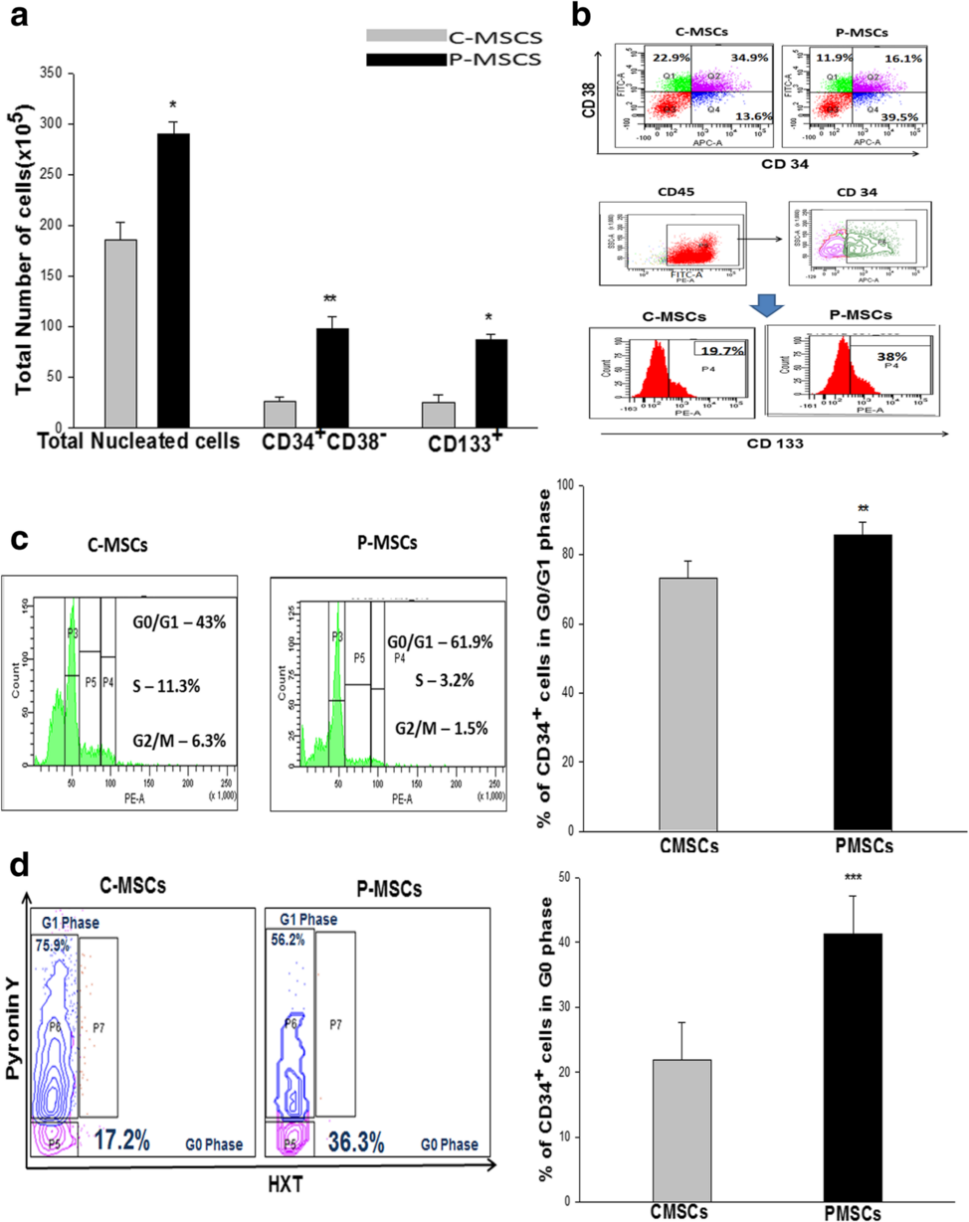
P-MSCs supported robust expansion of CD34^+^ cells without deterring their quiescence state. **a** Co-culturing of CD34^+^ cells with P-MSCs harbored a significantly higher TNC yield, CD34^+^CD38^−^ and CD133^+^ cells than C-MSCs as feeders. **b** *Upper panel*- A representative FACS dot plot showing higher CD34^+^CD38^−^ cells with P-MSCs as feeders. *Lower panel*- A representative FACS histogram depicting total CD34^+^CD133^+^ cells in the co-culture C- and P MSCs as feeders. **c** Cell cycle analysis demonstrated an increase of CD34^+^ cells at G0/G1 phase with P-MSCs as feeders and higher numbers of cycling CD34+ cells in cultures comprised of C-MSCs as feeders. **d** FACS dot plot profile of Hoechst and PyroninY based cell cycle analysis showing P-MSCs as feeders had a higher percentage of CD34^+^ cells in G0 (quiescence) stage in the co-cultures with P-MSCs than C-MSCs. Data are represented as mean ± standard deviation from three independent sets of experiments. *p ≤ 0.05, **p ≤ 0.01, and ***p ≤ 0.001 *P-MSCs* placenta-derived mesenchymal stem cells, *C-MSCs* cord-derived mesenchymal stem cells, *FACS* fluorescence-activated cell sorting

Equal numbers of the CD34^+^cells from a single cord blood unit were seeded separately on pre-established irradiated C- and P-MSCs obtained from the same donor and were cultured for ten days. CD 34^+^ cells expanded on P-MSCs (P-MSCs:CD34^+^ co-cultures) displayed a significant (two-fold) increase in total nucleated cell (TNC) yield, a 2.5-fold increase in immature hematopoietic progenitors (CD34^+^CD38^−^) and a higher proportion of the most primitive CD133^+^ cells compared to those expanded with C-MSCs (C-MSCs:CD34^+^ co-cultures) (Fig. 2a). Figure 2b upper panel is a dot plot representation which shows an increase in the CD34^+^CD38^−^ cells (39.5 %) in the P-MSCs:CD34^+^ co-cultures while C-MSCs:CD34^+^ co-cultures harbored committed cells CD34^+^CD38^+^ (34.9 %). The histogram of CD133 expression on CD45^+^CD34^+^ cells from a representative sample is shown in Fig. 2b (lower panel).

To further validate our observation we compared the hematopoietic supportive function of C-MSCs and P-MSCs with that of the gold standard, i.e., BM-MSCs. BM-MSCs were thus used as a positive control. BM-MSCs used in these experiments were first characterized morphologically, phenotypically, and by studying their differentiation ability towards three lineages, i.e., osteo, adipo, and chondro. They were found to satisfy the criteria laid down by ISCT to be identified as MSCs (see Additional file 1: Figure S1a and b). When the three types of MSCs were compared for their ability to expand UCB HSCs, it was observed that P-MSCs though not superior to BM-MSCs, were closer to them. On the contrary, C-MSCs were found to be the least efficient in expanding UCB HSCs. This was evident from higher expansion of the CD34^+^ CD38^−^ sub-population and the most primitive CD133^+^ fraction (see Additional file 1: Figure S1c), as well as from their higher clonogenicity (see Additional file 1: Figure S1d) when the CD34^+^ cells were expanded on BM-MSCs and P-MSCs as compared to C-MSCs.

#### P-MSCs expanded HSCs without hindering their quiescence state

Since P-MSCs had resulted in the massive expansion of HSCs, we next checked if the expansion was caused at the expense of exhausting the stem cell pool. A significantly higher percentage of CD34^+^ cells were in the G0/G1 phase in P-MSCs:CD34^+^ co-cultures as compared to the C-MSCs set (Fig. 2c). The left panel of Fig. 2c shows a representative FACS profile of PI based cell cycle wherein an increase in the cycling HSCs (as represented by the S/G2M phase of the cell cycle) is seen in C-MSCs:CD34^+^ co-cultures. Figure 2d depicts a representative FACS profile of Hoechst and PyroninY based resolution of the G0/G1 phase of the cell cycle, where P-MSCs:CD34^+^ co-cultures showed that 37 % of the CD34^+^ cells were in the G0 phase as opposed to 17 % in C-MSCs:CD34^+^ co-cultures. This observation clearly indicates that P-MSCs supported proliferation of CD34^+^ cells without compromising its quiescence state.

### CD34^+^ cells expanded with P-MSCs exhibited augmented *in vitro* functional attributes

**Fig. 3:**
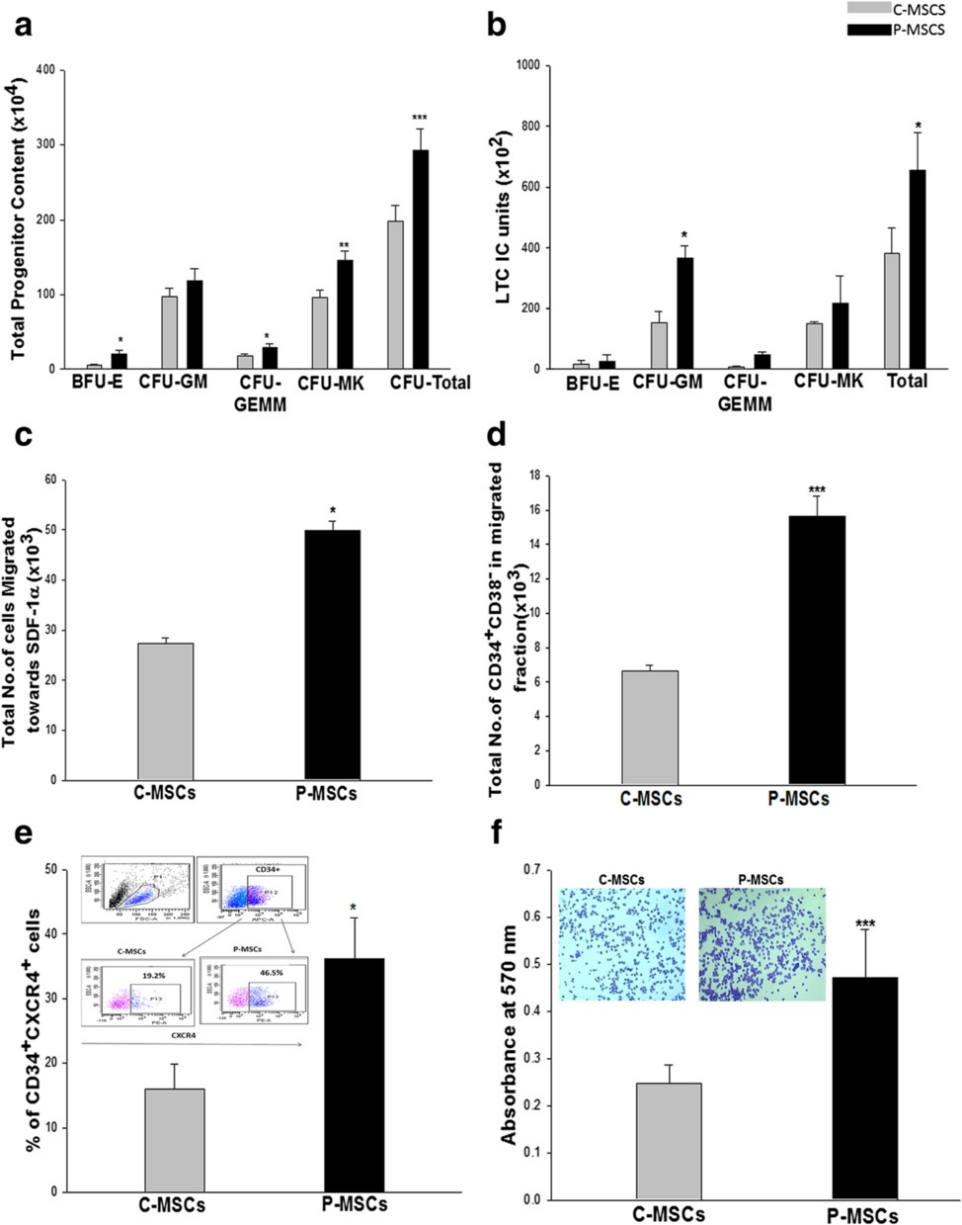
CD34^+^ cells expanded on P-MSCs have augmented *in vitro* functionality of the expanded cells. **a** P-MSCs:CD34+ co-cultures displayed higher blast-forming unit erythroid (BFU-E), granulocyte–monocyte (GM), granulocyte-erythroid-monocyte megakaryocyte (GEMM), and megakaryocytes (MK) colonies when compared with C-MSCs as feeders. **b** P-MSCs as feeders maintained significantly higher LTC-IC units than C-MSCs. **c** Trans well migration assay displayed significantly augmented migration of the expanded cells towards SDF-1α in the P-MSCs set as compared to the C-MSCs set. **d** Migrated fraction from the co-culture with P-MSCs revealed significantly higher numbers of primitive CD34^+^CD38^−^cells. **e** Superior migratory response might be attributed to the higher percentage of CD34^+^CXCR-4^+^ cells in co-cultures with P-MSCs as feeders. **f** Quantitation of the attachment to the fibronectin was carried out after lysing the adhered cells and measuring color intensity at 570 nm. The *inset* shows sort-purified CD34^+^ expanded on P-MSCs adhered to fibronectin in higher numbers. Data are represented as mean ± standard deviation from three independent sets of experiments. *p ≤ 0.05, **p ≤ 0.01, and ***p ≤ 0.001. *P-MSCs* placenta-derived mesenchymal stem cells, *C-MSCs* cord-derived mesenchymal stem cells, *LTC-IC* long term culture initiating cell

Owing to the fact that C- and P-MSCs co-cultures presented differences in the phenotype of the expanded population, we next determined their functionality. P-MSCs:CD34^+^ co-cultures showed greater clonogenicity as evaluated by CFU assay and manifested significantly higher BFU-E (four-fold), CFU-GEMM (1.6-fold), CFU-MK (1.5-fold), and total CFU (1.5-fold) as compared to C-MSCs:CD34+ co-cultures (Fig. 3a).

We further analyzed the self-renewal capacity of expanded cells by the LTC-IC assay. Both co-cultures revealed the formation of differential colonies in the LTC-IC assay. However, P-MSCs:CD34^+^ co-cultures harbored significantly higher total LTC-IC units as compared to C-MSCs:CD34^+^ co-cultures (Fig. 3b).

Trans-well migration assay is an *in vitro* method to ensure chemotaxis of expanded cells towards SDF-1α which is an important step for their successful lodgment in the BM. Cells expanded on P-MSCs showed a significantly higher number of migratory cells than cells expanded on C-MSCs (Fig. 3c). Upon analysis of the migrated fraction it was observed that the migrated fraction of P-MSCs:CD34^+^ co-cultures had approximately two-fold more primitive CD34^+^CD38^−^cells than cells co-cultured with C-MSCs (Fig. 3d). This augmented chemotactic response was attributed to the significantly higher percentage of CD34^+^CXCR-4^+^ population in the P-MSCs:CD34^+^ co-cultures than in the C-MSCs:CD34+ co-cultures (Fig. 3e). A representative FACS profile is shown as an inset in Fig. 3e.

After transplantation, HSCs establish themselves in the microenvironment via adhesive interactions with cellular and ECM components. We next checked their ability to adhere to fibronectin *in vitro*. Cells expanded on P-MSCs exhibited significantly superior adhesion to fibronectin as evident from the higher number of crystal violet stained cells seen attached to fibronectin (Fig. 3f inset), as compared to the cells expanded on C-MSCs. The attachment was also quantified by lysing the cells and measuring the absorbance at 550 nm (Fig. 3f).

### Co-culturing of CD34^+^ cells with P-MSCs exhibited enhanced SCID repopulation ability

#### Primary engraftment

**Fig. 4:**
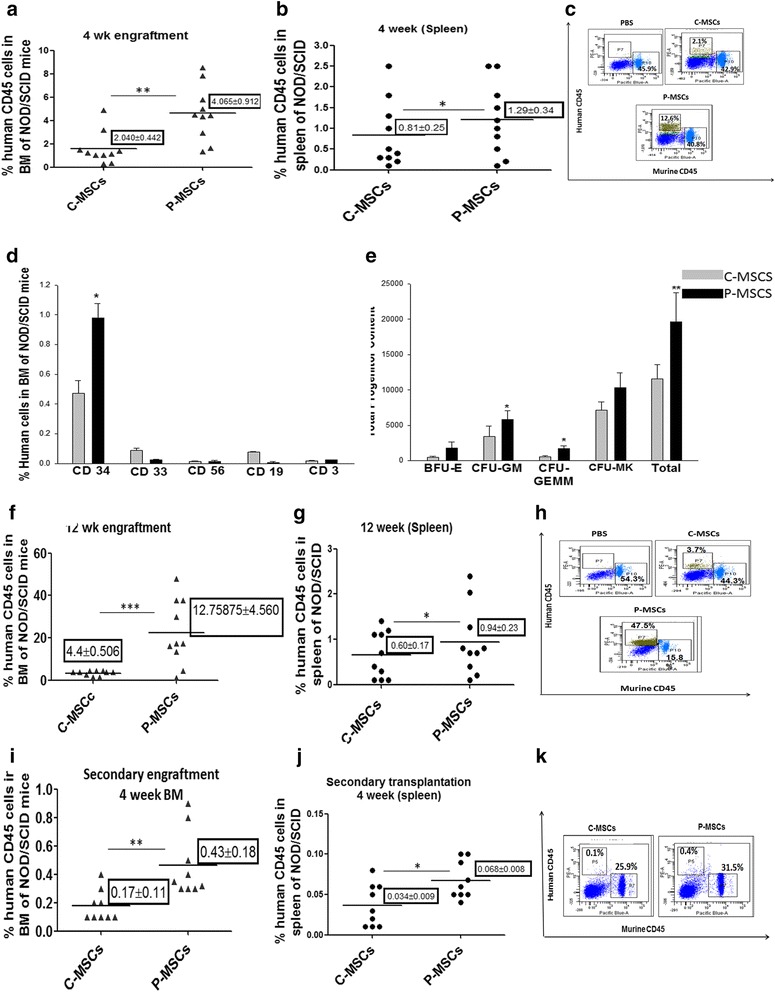
P-MSCS imparts significantly higher SCID repopulating ability on expanded CD34^+^ cells than C-MSCs. **a**,**b** At four weeks post transplantation (short term engraftment) the bone marrow(a) and spleen(b) of the mice receiving cells expanded on P-MSCs showed a significantly higher percentage of human CD45^+^ cells. Each dot in the graph represents an individual animal. **c** Representative FACS dot plots showing a higher percentage of human CD45^+^ cells in the BM of NOD/SCID mice infused with cells expanded on P-MSCs on the background of murine CD45^+^ cells. **d** Donor derived7 multi-lineage engraftment was seen in the BM of NOD/SCID mice for P- and C-MSCs expanded sets as determined by CD34 (stem cells), CD33 (myeloid cells), CD56(NK cells), CD19 (B cells), and CD3 (T cells). **e** Donor-derived committed progenitors were higher in the BM of mice receiving cells expanded on P-MSCs than on C-MSCs as evaluated by performing an *in vitro* colony formation assay on MBM using human specific growth factors. **f**, **g** Long term engraftment (12 weeks) indicated significantly higher chimerism (% human CD45) was detected in the BM (f) and spleen (g) of NOD/SCID mice receiving cells from P-MSCs co-cultures. **h** Representative FACS dot plots showing higher percentage of human CD45^+^ cells in BM of NOD/SCID mice infused with cells expanded on P-MSCs in the murine CD45^+^ background after 12 weeks. **i**, **j** Serial transplantation assay showed a significantly higher percentage of human CD45^+^ in BM (i) and spleen (j), the secondary recipient receiving cells from mice infused with cells expanded on P-MSCs. **k** Representative FACS dot plots showing a higher percentage of human CD45^+^ cells in the BM of NOD/SCID mice infused with cells from primary recipient. Data are represented as mean ± standard deviation from ten different mice. (n = 10) *p ≤ 0.05, **p ≤ 0.01, and ***p ≤ 0.001. *P-MSCs* placenta-derived mesenchymal stem cells, *C-MSCs* cord-derived mesenchymal stem cells, *FACS* fluorescence-activated cell sorting, *BM* bone marrow

Expanded cells (10^6^) were infused into sub-lethally irradiated NOD/SCID mice and four weeks after infusion short term engraftment in the BM was assessed by probing human CD45 cells against the host murine CD45 background. An irradiated mouse infused with PBS was used as a negative control. Cells expanded on P-MSCs showed significantly higher chimerism in the BM and spleen of NOD/SCID mice than those expanded on C-MSCs (Fig. 4a and b). Representative FACS dot plots, revealing a higher percentage of HuCD45^+^ cells in the BM of NOD/SCID mice infused with cells expanded on P-MSCs, are shown in Fig. 4c. The multi-lineage engraftment was confirmed by examining the donor-derived CD34 population, myeloid cells (CD33), and lymphoid cells (CD3, CD19, and CD56) in the BM of recipient mice. No predisposition for any specific lineage was observed (Fig. 4d). Further donor-derived committed progenitors were evaluated by an *in vitro* colony formation assay on MBM using human specific growth factors. A significantly higher CFU content was found in the BM of mice that received cells from P-MSCs:CD34^+^ co-cultures after four weeks (Fig. 4e).

To ensure the long term engraftment potential, the presence of human CD 45^+^ cells was also evaluated in the MBM after 12 weeks. Approximately three- and 1.5-fold higher chimerism was detected in BM and spleen, respectively, with the cells from P-MSCs:CD34^+^ co-cultures than with the cells from C-MSCs:CD34^+^ co-cultures (Fig. 4f and g). A FACS profile depicting the same is shown in Fig. 4h. It should be noted that the percentage of huCD45 increased in the MBM after 12 weeks owing to the *in vivo* proliferation of the infused cells. The engrafted cells were able to undergo multi-lineage differentiation as shown in Additional file 2: Figure S2a. Significantly higher human CFC content was obtained with P-MSCs:CD34^+^ co-cultures as quantified and depicted in Additional file 2: Figure S2b.

#### Secondary transplantation

A serial transplantation assay was carried out to ensure the *in vivo* self-renewal of co-cultured HSCs. For that, BM cells from the primary recipient were harvested 12 weeks post transplantation and 10^5^ sort-purified huCD45 were re-infused into sub-lethally irradiated secondary recipient NOD/SCID mice. Four weeks post transplantation, cells from the P-MSCs:CD34^+^ co-cultures gave rise to 2.5-fold and two-fold higher chimerism in BM and spleen, respectively, than with C-MSCs as feeders (Fig. 4i and j). A representative FACS profile is depicted in Fig. 4k. The engrafted cells retained their multi-lineage differentiation potential as depicted in Additional file 2: Figure S2c which was further confirmed by quantitating human derived CFC in MBM by the CFU assay, as shown in Additional file 2: Figure S2d.

### P-MSCs supported survival of CD34^+^ cells via up-regulation of anti-apoptotic BCl-2 protein

**Fig. 5:**
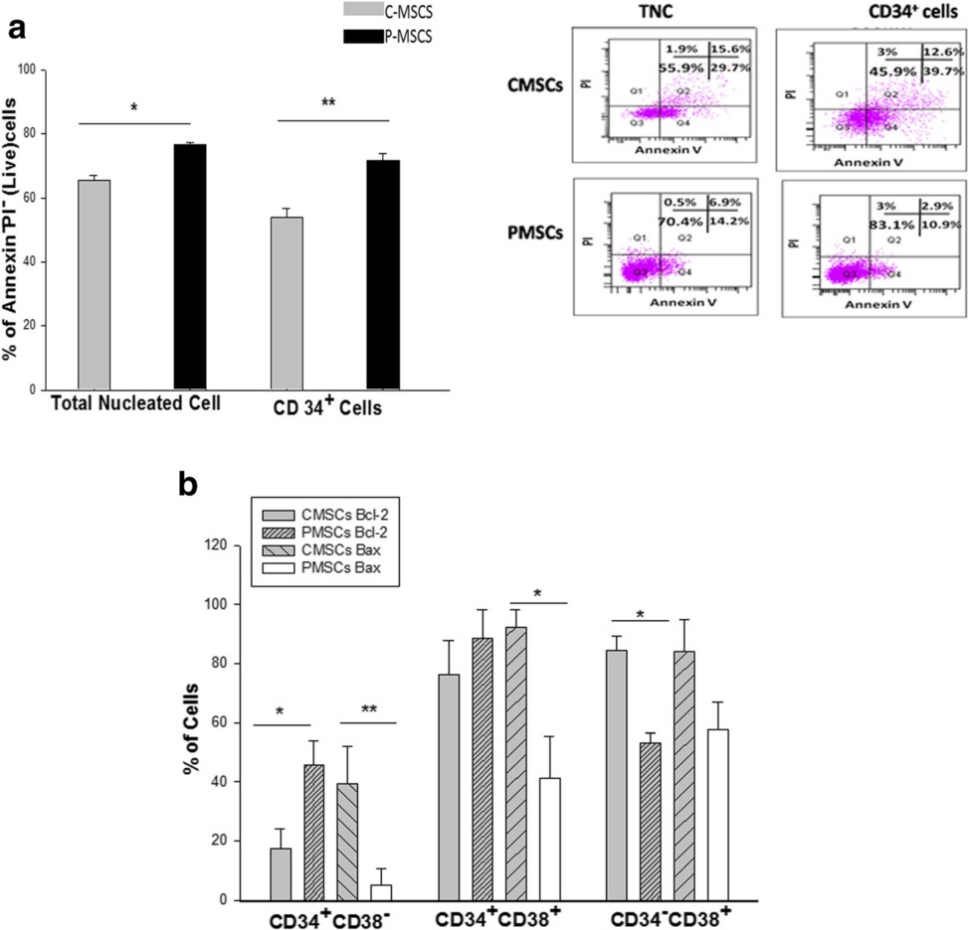
Co-culturing of CD34^+^ cells with P-MSCs reduced the level of apoptosis and expansion of the primitive HSCs by preventing apoptosis via up regulation of BCL-2. **a** Higher percentage of live TNC as well as gated CD34^+^ cells were detected in P-MSCs:CD34^+^ co-cultures. *Inset* depicts representative FACS profile depicting the same P-MSCs:CD34^+^ co-cultures (*Lower panel*), C-MSCs:CD34^+^ co-cultures (*Upper panel*). **b** Significantly higher levels of Bcl-2 were detected in CD34^+^CD38^−^ cells from the P-MSCs:CD34^+^ co-cultures. Co-cultures of C-MSCs displayed higher Bcl-2 levels in CD34^+^CD38^+^ and CD34^−^CD38^+^ cells. Bax expression was significantly higher in all the subsets from C-MSCs: CD34+ co-cultures. Data are represented as mean ± standard deviation from three different independent experimental sets. *p ≤ 0.05, **p ≤ 0.01, and ***p ≤ 0.001. *P-MSCs* placenta-derived mesenchymal stem cells, *TNC* total nucleated cells, *FACS* fluorescence-activated cell sorting, *C-MSCs* cord-derived mesenchymal stem cells

We next determined if the above observed improvement was due to a difference in the survival of the cells expanded with the two feeders. A significant reduction was observed in the apoptosis in the P-MSCs:CD34^+^ co-cultures as opposed to that with C-MSCs:CD34^+^ co-cultures. A significantly higher percentage of annexinV^−^PI^−^cells (viable cells) were observed in the total (1.2-fold) and CD34^+^ (1.5-fold) compartment in the P-MSCs:CD34^+^ co-cultures than in the C-MSCs:CD34^+^c o-cultures (Fig. 5a). Figure 5b depicts the FACS profile of a representative sample.

In order to study the mechanism behind this differential survival, the expression of BCl-2 and Bax was evaluated. It was found that P-MSCs had significantly up-regulated BCl-2 expression in the primitive (CD34^+^CD38^−^) (57.34 ± 3.45 %) and progenitor (CD34^+^CD38^+^) (~95 %) populations, while relatively low expression was observed in the totally differentiated cells (CD34^−^CD38^+^) (52.32 ± 4.23 %). In sharp contrast, C-MSCs showed the highest expression of BCl-2 in totally differentiated cells (≥95 %) and much lower expression in the primitive (17.98 ± 3.76 %) and progenitors (79.12 ± 1.45 %) populations. Conversely, P-MSCs:CD34^+^co-cultures demonstrated significantly lower levels(≤60 %) of Bax protein in the primitive population (5.25 ± 1.25 %), progenitors (41.06 ± 3.69 %), and totally differentiated cells (59.76 ± 5.45 %), while the Bax expression was higher in the primitive population (38.54 ± 5.85 %), progenitors(94.23 ± 2.15 %), and totally differentiated cells (90.56 ± 2.55 %) with C-MSCs as feeders (Fig. 5c). It should be noted that the percentage of Bax^+^CD34^+^CD38^−^ was negligible in the cells expanded on P-MSCs.

We have earlier reported that the addition of anti-apoptotic agents resulted in increased expansion efficiency of suspension cultures of UCB CD34^+^ cells [17]. Since higher levels of apoptosis were detected in the C-MSCs:CD34^+^ co-cultures, we checked if this system could be made equivalent to the P-MSCs co-cultures by the addition of anti-apoptotic molecules. Addition of the pan-caspase inhibitor zVADfmk (500nM) and the calpain-1 inhibitor zLLYfmk (15 μM) significantly increased the yield of TNC (see Additional file 3: Figure S3a) and primitive cells (see Additional file 3: Figure S3b) compared to those without inhibitors. Also, an improvement was seen in the functionality of the cells expanded with inhibitors as assessed by the CFU assay (see Additional file 3: Figure S3c), *in vitro* migration towards SDF1α (see Additional file 3: Figure S3d) and *in vitro* adhesion to fibronectin (see Additional file 3: Figure S3e).

### Physical interaction between P-MSCs and CD34+ cells is indispensable for the beneficial effect of P-MSCs

Next we checked if the modulatory effect of P-MSCs was contact or cytokines dependent. A significant reduction (≥50 %) was observed in the TNC yield (see Additional file 4: Figure S4a), the total CD34^+^ content, and primitive content (see Additional file 4: Figure S4b) in non-contact cultures as compared to contact cultures of P-MSCs with CD34^+^ cells. However, non-contact co-cultures of C-MSCs showed a marginal reduction in cell yield (see Additional file 3: Figure S3a). The viability of the cells in the non-contact cultures of P-MSCs was greatly compromised, as evident from a significant reduction in the percentage of total live (48.62 %) as well as CD34^+^ cells (38.67 %) (see Additional file 4: Figure S4c). On the contrary, no change in the viability of non-contact C-MSCs culture was observed (data not shown). Thus, physical contact with the P-MSCs is a key for the maintenance of HSCs.

To identify the molecular basis of these interactions, we carried out a series of blocking experiments. Out of the myriad of interacting molecules, we chose to focus on integrin (α4β1/VCAM-1 and α5β1/fibronectin) and SDF-1α/CXCR-4 since they are involved in providing survival signals to HSCs in the niche. Blocking of VLA-4(α4β1), VLA-5(α5β1), and CXCR-4 on CD34^+^ cells was done singly or in combination with the blocking antibodies. The unblocked/blocked cells were then co-cultured with respective stroma for 96 hrs and the proliferation was assessed by MTT. Individual blocking of the integrins had minimal effect on the survival of CD34^+^ cells in the co-cultures with C-MSCs and P-MSCs (see Additional file 4: Figure S4d,e). Blocking with individual or combinations of antibodies had no effect on the proliferation (see Additional file 4: Figure S4d), clonogenic ability (see Additional file 4: Figure S4f) and survival (see Additional file 4: Figure S4h) of the cells expanded on C-MSCs. In P-MSCs:CD34^+^ co-cultures, however, all the above parameters were compromised, particularly upon blocking integrins in combination with CXCR-4 (see Additional file 4: Figure S4e, g.i).

The results clearly indicate that P-MSCs exert their effect partly via integrin and SDF-1α mediated axis.

### Enhanced expansion on P-MSCs can be attributed to the functional differences between the two feeders

**Fig. 6:**
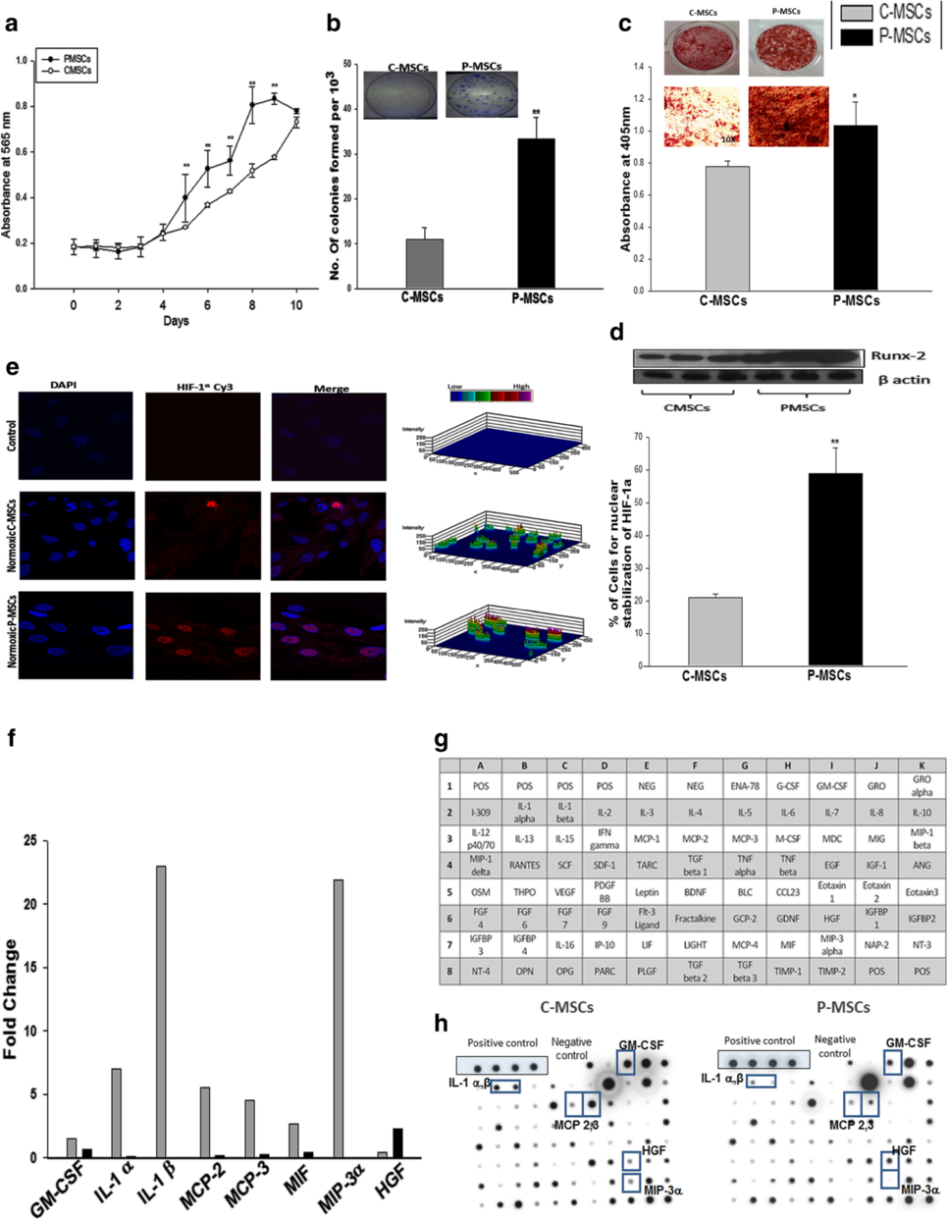
C-MSCs and P-MSCs differ in the functional attributes relevant to expansion of HSCs. **a** P-MSCs had higher rate of proliferation than C-MSCs as determined by MTT assay. **b** P-MSCs exhibited higher clonogenecity than C-MSCs even at lowest cell concentration. *Inset* depicts the representative CFU-F colonies stained with crystal violet. **c** P-MSCs exhibits superior differentiation to osteoblasts depicted by the calcium deposits stained with alizarin red S (*Left panel*). *Right panel* shows quantitative analysis after extraction of the dye and its quantitation at 405 nm. **d** Superior osteoblastic differentiation may be due to the pre-osteoblastic nature of P-MSCs by virtue of higher RUNX-2 expression. **e** Higher normoxic stabilization of HIF-1α in the nucleus of the P-MSCs than C-MSCs. *Left panel* shows 3D representations of expression of the HIF-1α as 3-D histograms. Percentage of the positive nuclei from ten random fields/slide of three independent experiments is represented in the graph. **f** Fold change in the cytokines expression by C-MSCs and P-MSC, respectively. Data represented as mean value of two independent paired samples. **g** C-MSCs had a distinctive secretion profile mainly of pro-inflammatory cytokines as checked by membrane-based cytokine array. **h** Represents the hybridization results for a representative sample. Data are represented as mean ± standard deviation from three different independent experimental sets. *p ≤ 0.05, **p ≤ 0.01, and ***p ≤ 0.001. *P-MSCs* placenta-derived mesenchymal stem cells, *C-MSCs* cord-derived mesenchymal stem cells, *HSCs* hematopoietic stem cells, *CFU-F* colony forming unit-fibroblasts

With P- and C-MSCs having exhibited differences in the hematopoiesis supportive function, we further investigated the indigenous differences between these two stromal populations. P-MSCs showed a significantly higher rate of proliferation than C-MSCs (Fig. 6a). P-MSCs retained significantly higher clonogenecity even at low cell count (10^3^) cells as compared to C-MSCs (Fig. 6b and inset). C- and P-MSCs could be differentiated into osteoblasts, adipocytes, and chondrocytes. However, qualitatively and quantitatively P-MSCs displayed greater osteogenic potential as compared to C-MSCs (Fig. 6c left panel). To corroborate these findings, we further checked the levels of RUNX-2 in P-MSCs and C-MSCs. It was found that the expression of RUNX-2 was three-fold higher in P-MSCs (Fig. 6d).

Some of the attributes of P-MSCs, such as predisposition to the osteoblastic lineage and higher RUNX-2 expression, were found to be similar to MSCs found in the endosteum, where hypoxia leads to the stabilization of HIF-1α which is a key regulator of hematopoiesis. Therefore, we checked if there was any difference in HIF-1α level in both MSC populations. P-MSCs showed significantly higher normoxic stabilization of HIF-1α than C-MSCs. Fig. 6e shows the confocal images of the nuclear stabilization of HIF-1α in C- and P-MSCs under normoxic conditions. No difference was observed for hypoxic stabilization of HIF-1α in C and P-MSCs (data not shown).

The balance between differentiation and maintenance of the stem cells is regulated by complex molecular mechanisms involving soluble factors and cell-cell interactions. Therefore, we compared the secretory profile of C-MSCs and P-MSCs using a semi-quantitative cytokine array (Fig. 6g). The majority of the cytokines secreted by C-MSCs were of a pro-inflammatory nature with a more than two-fold up-regulation of IL-1α, IL-1β, MCP-2, MCP-3, and MIP-3α observed, whereas the quantities of these cytokines were negligible in the CM of P-MSCs except for HGF (Fig. 6f and h). The graph represents a quantitative analysis of two independent paired samples.

### P-MSCs displayed higher Wnt activation resulting in improved Notch signaling in the expanded HSCs

**Fig. 7:**
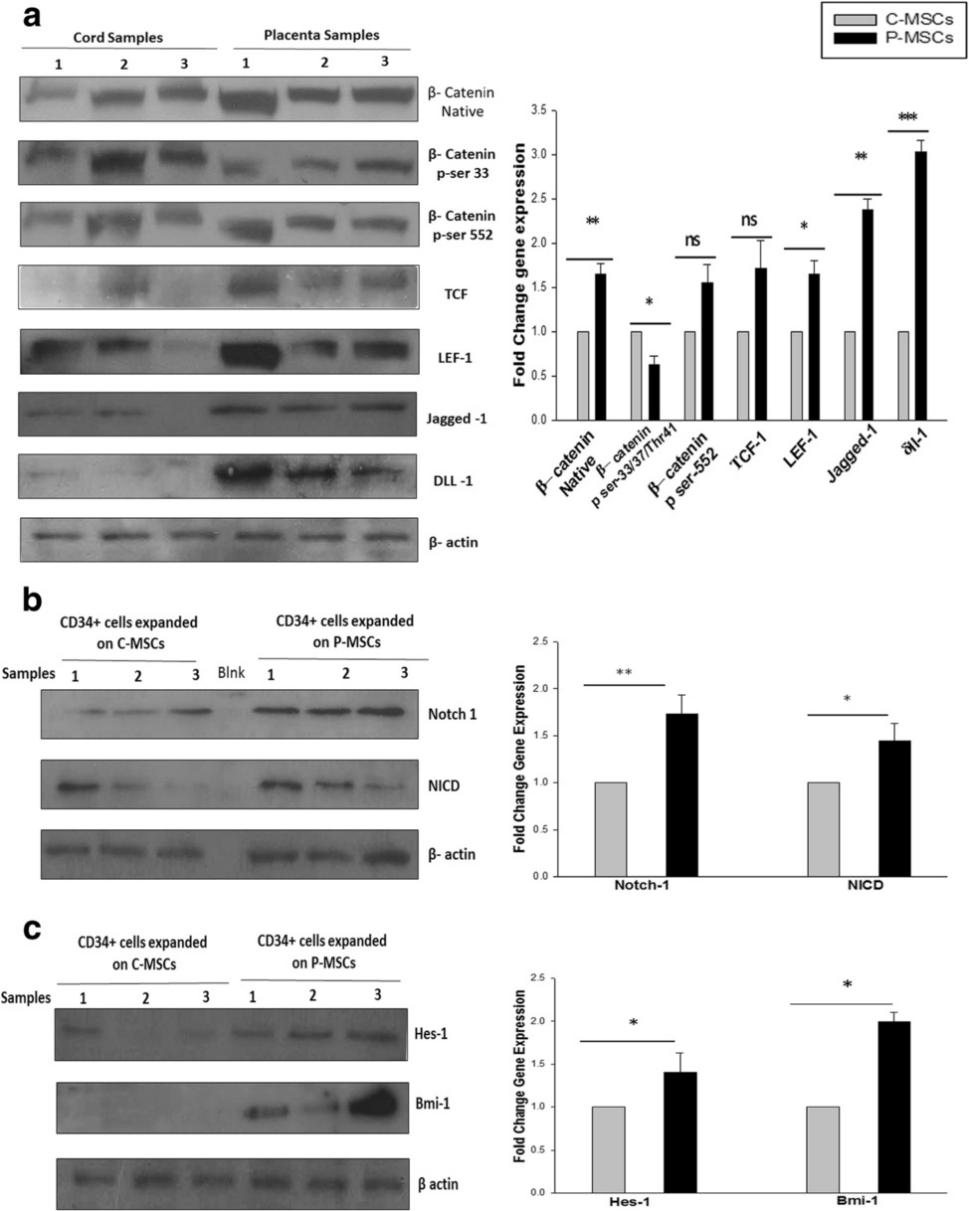
P-MSCs displayed higher Wnt activity leading to collateral increase into Notch signaling in the expanded CD34^+^cells. **a** Higher expression of native β-catenin. Phosphorylated β-catenin (ser-552) was found in the P-MSCs. Higher expression of phosphorylated β-catenin (ser-33/34Thr41) in C-MSCs. The expression of downstream targets of Wnt, TCF, LEF-1, jagged-1 and δ-like one (dll-1) was higher in P-MSCs than C-MSCs. The graph represents the fold change in the expression after normalization to β-actin. **b** Sort purified CD34^+^ cells from P-MSCs. Co-cultures showed significant up regulation of Notch I and intracellular domain of Notch (NICD). The quantitation of the same is represented as a graph. **c** The CD34^+^ cells expanded on the P-MSCs exhibited higher expression of Hes-1 and Bmi-1, downstream target of Notch, quantitatively represented as a graph. Data are represented as mean ± standard deviation from three different samples. *p ≤ 0.05, **p ≤ 0.01, and ***p ≤ 0.001. *P-MSCs* placenta-derived mesenchymal stem cells, *C-MSCs* cord-derived mesenchymal stem cells

To elucidate the molecular mechanism behind the better hematopoiesis observed in P-MSCs:CD34^+^ co-cultures, we evaluated the levels of the Wnt and Notch signaling pathways, which are linked to self-renewal and maintenance of HSCs. We observed a 1.5-fold higher expression of β-catenin, an effector of in irradiated P-MSCs than in irradiated C-MSCs (Fig. 7a). Further, phosphorylation at the ser-33/37/Thr41 residue which is linked to degradation of β -catenin mediated by GSK-3β was found to be reduced in the P-MSCs. The level of activator phosphorylation at residue ser-552 by AKT/PKA was also found to be 1.3-fold higher in P-MSCs. A downstream target of the canonical β–catenin pathway, the levels of TCF were found to be 1.5-fold higher in P-MSCs stromal cells. Further, promoters of jagged-1 and δ-like one (dll-1), also shown to be targets of β-catenin, were found to be up-regulated in P-MSCs (Fig. 7a). Therefore, we investigated the corresponding activation of notch signaling in the HSCs expanded in the co-culture. Figure 7b shows significantly higher expression of total Notch 1 and cleaved Notch 1(NICD form) in the CD34^+^ cells expanded on P-MSCs (1.5-fold). The downstream targets of Notch, Bmi-1 and Hes-1 were also found to be up-regulated 2.5-fold and 1.2-fold, respectively, in the P-MSCs:CD34^+^ co-culture (Fig 7c). Collectively, these results indicate that higher levels of Wnt/β-catenin signals in the P-MSCs resulted in the activation of Notch signaling in the CD34^+^ cells.

## Discussion

*Ex vivo* expansion of UCB HSCs is attempted to increase the cell dose for HSCs transplantation in adult patients [1, 4, 5]. HSCs are either expanded in suspension cultures or in support with the different cell types from the hematopoietic niche [6, 8–10]. Among the different cell types used for expansion of HSCs, MSCs have been found to be the most appropriate feeders [4]. Although BM remains the chief source of MSCs, obtaining this source involves an invasive procedure, thus posing a risk to the donor. The quality of MSCs is also influenced by the age of donors. On the contrary, the fact that MSCs from neonatal tissues can be isolated with ease, with no harm to the donor, and their primitive nature indisputably poses an attractive alternative to BM-MSCs. Thus, perinatal tissues may be ideal sources for cell therapy [13]. C- and P-MSCs are being extensively studied with reference to their immune modulatory functions and trans-differentiation potential and have proven to be better than BM-MSCs [14]. C-MSCs are further seen as a promising alternative to BM-MSCs as feeders for expanding HSCs [18, 19]. However, the potential of P-MSCs as feeders for HSCs expansion is probably underestimated while it is being explored as a valuable source for cell replacement therapies. Despite extensive comparison of MSCs derived from these tissues with the BM-MSCs, to date none of these studies have directly compared the hematopoietic supportive function of C-MSCs with that of P-MSCs. We found striking differences in their ability to serve as feeders, in that P-MSCs were found to be better feeders than C-MSCs for the support of *ex vivo* hematopoiesis. Furthermore, our observation with the BM-MSCs as positive control additionally strengthens the inference that P-MSCs are equally as competent as BM-MSCs for supporting HSC expansion as opposed to C-MSCs. However, since our primary aim here was to compare C- and P-MSCs, the detailed mechanism underlying the difference in the ability to support hematopoiesis was studied with respect to C- and P-MSCs only and not with BM-MSCs.

C-MSCs and P-MSCs were found to be morphologically and phenotypically similar but it was observed that P-MSCs could be cultured over 11–15 passages, as opposed to C-MSCs that could be maintained for only seven to eight passages (data not shown). P-MSCs were found to be sturdier as they sustained the transition from FBS-containing medium to the xenofree medium consisting of cord blood derived plasma better than C-MSCs [15]. These advantages make P-MSCs undoubtedly better and more convenient to maintain in cultures than C-MSCs. The superiority of P-MSCs for supporting the expansion of functionally superior and transplantable HSCs was established from the results of an array of *in vitro* and *in vivo* assays. P-MSCs promoted better expansion in terms of primitive progenitor content, CFU and LTC assays with improved adhesive and migratory properties. The results of *in vivo* assays, such as short/long term and secondary engraftment in the NOD/SCID mouse model, left little doubt that P-MSCs as feeders are more efficient at expansion of UCB HSCs than C-MSCs.

While optimizing the co-culture system, we opted to select irradiated MSCs as feeders for expansion of HSCs. In a clinical scenario, prior to HSC transplantation, patients usually undergo myeloablation in the form of radiation therapy. The transplanted HSCs then lodge on the irradiated stroma, proliferate, and establish hematopoiesis. Thus, irradiated stroma probably stimulates the HSCs. Taking this fact into consideration, along similar lines, we also compared the effect of unirradiated and irradiated MSCs on their capacity to support hematopoiesis and found that indeed irradiated MSCs had substantially increased efficiency to expand HSCs (data not shown). Available literature also supported our observation. Celebi et al. [20] have reported that irradiated MSCs partly mimic the endosteal niche thereby supporting better expansion of HSCs *ex vivo*. Furthermore, Walenda et al. [21] highlights the maintenance of primitive CD34^+^CD38^−^ cells after culturing on irradiated bone marrow MSCs.

Next, in an attempt to seek the differences in the characteristics of the two feeders that regulate the hematopoietic supporting ability, it was found that P-MSCs robustly supported the expansion of primitive HSCs, as opposed to C-MSCs, that supported the expansion of cells dominant in CD34^+^CD38^+^ and CD34^−^CD38^+^ progenitor cells. The observed expansion of different subsets in the two co-culture systems could be attributed to the differences in cytokines secreted by C- and P-MSCs. C-MSCs secrete a variety of the pro-inflammatory cytokines, such as IL-1α, β, GM-CSF and MCP-2, 3, driving differentiation of HSCs [22], thus validating the observed expansion of the progenitor population and decline in the primitive HSCs content in co-cultures of C-MSCs. In sharp contrast, the P-MSCs secreted negligible levels of the above cytokines. Cytokines secreted by both the MSCs were similar to those reported in the literature for other MSCs, such as BM-MSCs, adipose MSCs, etc. [23, 24]. Still, we find striking differences in the levels of their secretion when we compared C- and P-MSCs. Further, Fong et al. [25] have reported better expansion of UCB CD34^+^ cells with the conditioned medium obtained from Wharton’s jelly-derived MSCs (WJ-MSCs) than when co-cultured with MSCs. This clearly suggests that CM of WJ-MSCs plays a role in the expansion of HSCs even in the absence of the feeder cells themselves, which supports our observation about the cytokines secreted by C-MSCs and its effect on the expansion of committed progenitors.

The improved expansion in co-cultures of P-MSCs, however, cannot solely be attributed to the presence of HGF, whose levels were merely two-fold higher in P-MSCs, as against the levels of C-MSCs cytokines. Our data on contact and non-contact cultures of P-MSCs convincingly demonstrate that P-MSCs exert a beneficial effect only upon physical contact with the HSCs. Thus, we investigated the contribution of other cellular factors that might be responsible for the expansion of primitive HSCs. One such crucial factor in deciding the HSC fate is cell-cell contact, as explained by Da Silva et al. [26]. At endosteum, HSCs interact with MSCs/osteoblast predominantly via (SDF-1/CXCR-4) or integrin mediated interactions [26, 27]. Likewise, Fonseca et al. [28] reported a significant increase in the primitive CD34^+^CD38^−^ cells which were in close association with MSCs. They further demonstrated that the basis of physical contact between HSCs and MSCs was dominated by the SDF-1/CXCR-4 and integrin axis. We also report the dominance of the SDF-1α and integrin axis in our co-culture comprising P-MSCs by a series of blocking experiments further underscoring the supremacy and specificity of such interactions. Thus, we believe that P-MSCs utilize similar cellular interactions as BM-MSCs for maintenance of primitive HSCs. The observed differences in the expanded cells could also be due to the differential survival of these subsets as MSC-HSCs co-cultures exhibited reduction in apoptosis of HSCs [29]. We observed striking differences in the survival of cells expanded on C-MSCs and on P-MSCs due to contrasting levels of Bcl-2 and Bax in the different subsets. Higher Bcl-2 levels were found in the primitive subset leading to the persistence of this population in P-MSCs co-cultures. Higher Bcl-2 levels are not only responsible for the survival of HSCs but several investigators have established its link with *in vivo* functionality of HSCs [30, 31]. We indeed found higher primary and secondary engraftment of HSCs expanded on P-MSCs than on C-MSCs. The superior SRC potential of cells expanded on P-MSCs could be due to the higher migration towards SDF-1α observed with these cells *in vitro* which might have resulted in better migration to BM in irradiated NOD/SCID mice. Second, being physically attached to the HSCs the P-MSCs infused along with the graft might be responsible for better engraftment. This observation is supported by findings of Hiwase et al. [32] who reported better engraftment in mice when transplanted with P-MSCs along with HSCs. Probably the HSCs that are physically attached to P-MSCs are successfully delivered to the BM niche. Our observations between contact and non-contact cultures further strengthen this possibility. Moreover, P-MSCs were found to be more immature than C-MSCs as indicated by their higher proliferative potential and clonogenicity. This might be another reason for better engraftment of cells expanded on P-MSCs as this conjecture is supported by the findings of Yim et al. [33].

To understand the molecular basis of the above observed differences we considered the stromal cell mediated Wnt/β catenin signaling and its effects on the HSCs. Activation of Wnt/β-catenin signaling in stromal cells is shown to promote hematopoiesis by up-regulating the expression of the notch ligands (jagged-1, Dll-1) on stromal cells [34]. P-MSCs showed higher β-catenin levels which may have caused the higher expression of jagged − 1 and Dll-1 on their surface resulting in a corresponding increase in the notch receptors and its responsive genes in the CD34^+^ cells expanded on P-MSCs. We further tried to elevate the level of Wnt signaling in C-MSCs by lithium chloride treatment which resulted in a moderate improvement in the expansion of HSCs (data not shown). However, the contribution of the other signaling pathways is yet to be determined.

As a consequence of higher Wnt activity, MSC differentiation is skewed towards osteoblast over the other lineages [35]. Therefore, in an attempt to establish whether the observed differences in the two feeder cells might influence differentiation to osteoblasts, we checked the level of Runx-2 and found significantly higher expression in the P-MSCs. According to Chitteti et al. [36], primitive osteoblasts with high Runx-2 expression resulted in better promotion of HPC proliferation, additionally strengthening our observation of superior expansion of HSCs with pre-osteoblastic P-MSCs. Further, Runx-2 expressing pre-osteoblasts are predominantly found in the endosteum of the bones which is hypoxic in nature. To further confirm this, we checked the HIF-1α levels; there was no difference in the HIF-1a stabilization in the two feeders when maintained under hypoxic conditions (data not shown). We indeed found that P-MSCs showed significantly higher nuclear stabilization of HIF-1α under normoxic conditions, corroborating the reports by Palomaki et al. [37, 38]. Collectively, our data strongly suggest that P-MSCs partly provide conditions resembling the endosteal niche of the bone marrow. The similarity between the placenta and bone marrow could be explained by the fact that the placenta is a reservoir of HSCs during development [39], while the endosteum remains a reservoir of HSCs throughout life by maintaining steady state hematopoiesis establishing a solid link between bone marrow and placenta. However, we have not done direct comparisons of P-MSCs with BM-MSCs in this respect as this was not the main objective of this manuscript. Nonetheless, we found that P-MSCs have a similar capacity to sustain *ex vivo* hematopoiesis as that of BM-MSCs. Thus, P-MSCs appear to be **a** non-invasive and clinically equivalent substitute for BM-MSCs to be used as feeders for expansion of HSCs.

## Conclusions

In summary we report here that P-MSCs are more suitable as feeders than C-MSCs for expansion of UCB-derived HSCs. Higher activity of Wnt/Notch signaling, normoxic HIF-1α stabilization, pro-osteoblastic phenotype and HGF secretion along with a drastic reduction in apoptosis cumulatively add to the improved ability of HSCs expansion of P-MSCs. Current cell therapy protocols utilize C-MSCs as an alternative to BM-MSCs. However, our findings underscore the use of P-MSCs over C-MSCs for optimizing current protocols aimed at expanding HSCs. Further, since our HSC-MSC co-cultures were grown in serum free conditions, they hold potential to be directly translated in the clinical settings.

## Additional files

Additional file 1: Figure S1. BM-MSCs and P-MSCs had similar potential for expansion of CD34+ cells. **a** BM-MSCs have fibroblastic morphology, can be differentiated into osteoblasts, adipocytes and chondrocytes. **b** BM-MSCs were shown to exhibit expression of CD44, CD73, CD105, and CD90 and found not to express CD34, CD45, CD14, CD19, CD11b, and andHLA-DR. **c** BM-MSCs had robust expansion of total nucleated cells, CD34^+^CD38^−^, and CD133^+^ cells. **d** CD34^+^ cells expanded on BM-MSCs had superior clonogenic ability. Data are represented as mean ± standard deviation from four different samples. *p ≤ 0.05, **p ≤ 0.01, and ***p ≤ 0.001. (TIFF 5033 kb) Additional file 2: Figure S2. Cells expanded on P-MSCs gave rise to persistent engraftment in NOD/SCID mice. **a** Superior multi-lineage engraftment as denoted by donor-derived CD34 (stem cells), CD33 (myeloid cells), CD56 (NK cells), CD19 (B-cells), and CD3 (T-cells) after 12 weeks was detected in bone marrow of animals receiving cells from P-MSCs co-cultures as opposed to C-MSCs co-cultures (n = 10). **b** Mice infused with cells expanded on P-MSCs showed significantly higher numbers of donor-derived progenitors (CFU) after 12 weeks. **c** Multi-lineage engraftment in secondary recipient also showed superiority of the cells expanded on P-MSCs. (n = 9). **d** Secondary recipient similarly displayed a significantly higher number of human CFC in the mice receiving cells from primary recipient infused with cells from P-MSCs:CD34^+^ co-cultures. (n = 9). Data are represented as mean ± standard deviation from ten different mice. *p ≤ 0.05, **p ≤ 0.01, and ***p ≤ 0.001. (TIFF 2354 kb) Additional file 3: Figure S3. Addition of apoptotic inhibitors in C-MSCs:CD34^+^ co-cultures improved expansion of UCB CD34^+^ cells. **a** Addition of pan caspase inhibitor (zVADfmk) and calpain-1 inhibitor (zLLYfmk) to the C-MSCs:CD34^+^ co-cultures significantly improved total nucleated cell yield in comparison to the control co-cultures. **b** Significant increase in the numbers of CD34^+^CD38^−^ and CD133^+^ cells was detected in the co-cultures inclusive of zVADfmk and zLLYfmk as opposed to the ones without them. **c**,**d**,**e** Apoptotic inhibitors improved the *in vitro* functionality of the expanded cells as determined by CFU content (c), *in vitro* migration (d) and *in vitro* adhesion to fibronectin (e), respectively. Data are represented as mean ± standard deviation from three different independent experimental sets. *p ≤ 0.05, **p ≤ 0.01, and ***p ≤ 0.001. (TIFF 2676 kb) Additional file 4: Figure S4. P-MSCs favors the expansion of CD34^+^ cells in a contact dependent manner. **a** Significant reduction in total nucleated cells (TNC) was observed in non-contact cultures of P-MSCs while no effect was seen in non-contact cultures of C-MSCs. **b** Non-contact cultures of P-MSCs displayed reduction in the percentage of CD34^+^ and CD34^+^CD38^−^ cells as compared to contact cultures. **c** Reduction in the yield of TNC and CD34+ cells was due to an increase in the apoptosis in non-contact cultures as seen by a decrease in the annexinV^−^PI^−^ cells in non-contact cultures. **d** No change in the proliferation of CD34^+^ cells either singly blocked with CD49d (VLA-4), CD49e (VLA-5), and CXCR-4 or in combination with CD49d + CD49e and CD49d + CD49e + CXCR-4 was observed after co-culturing with C-MSCs for 96 hrs as assessed by MTT assay. **e** Drastic reduction was seen in the proliferation of CD34^+^ cells upon blocking with CD49d + CD49e, CXCR-4, and CD49d + CD49e + CXCR-4 and co-culturing with P-MSCs. **f** Clonogenecity of the cells expanded on C-MSCs remained unaltered even after blocking with anti-bodies. **g** Significant reduction was observed in the progenitor content after blocking the CD34^+^ cells with the combination of antibodies and co-culture on P-MSCs. **h** Percentage of live population remains unchanged in the blocked set as opposed to the unblocked set in the co-cultures comprised of C-MSCs. **i** Viability of the expanded cells blocked with integrins and CXCR-4 reduced significantly in co-cultures with P-MSCs as feeders. Data are represented as mean ± standard deviation from three different independent experimental sets. *p ≤ 0.05, **p ≤ 0.01, and ***p ≤ 0.001. (TIFF 4452 kb)

## Abbreviations

C-MSCs: Cord derived MSCs
HSCs: Hematopoietic stem cells
MSCs: Mesenchymal stem cells
P-MSCs: Placenta derived MSCs
P-MSCs: CD34+ co-cultures -CD34^+^ cells expanded on P-MSCs
C-MSCs: CD34^+^ co-cultures-CD34^+^ cells expanded on C-MSCs
